# Identifying dynamic antithrombin Ⅲ trajectories to predict clinical outcomes in intra-abdominal sepsis

**DOI:** 10.1016/j.jointm.2025.11.006

**Published:** 2026-01-20

**Authors:** Yuteng Ma, Yini Sun, Chaoyang Wang, Siang Huang, Qian Gao, Qianyi Xu, Mu Sun, Qiaojie Sun, Ming Dong

**Affiliations:** 1 Department of Gastrointestinal Surgery, The First Hospital of China Medical University, Shenyang, Liaoning, China; 2 Department of Critical Care Medicine, The First Hospital of China Medical University, Shenyang, Liaoning, China; 3 Population Health Sciences, University College London, London, UK; 4 Medical and Health Division, Clinical Specialty Product Center, Neusoft Corporation, Shenyang, Liaoning, China

**Keywords:** Intra-abdominal infection, Sepsis, Antithrombin III, Trajectory analysis, Mortality

## Abstract

**Background:**

Intra-abdominal infection (IAI) is the leading cause of sepsis and is often complicated by disseminated intravascular coagulation (DIC), leading to increased mortality. Antithrombin Ⅲ (AT Ⅲ), a crucial endogenous anticoagulant, becomes significantly depleted during sepsis due to increased consumption and reduced synthesis. Its levels are closely associated with disease severity and clinical outcomes. Currently, there is a lack of evidence on the dynamic changes of AT Ⅲ and their relationship with the severity and prognosis of sepsis caused by IAI.

**Methods:**

This was a prospective observational study. The patients with IAI-induced sepsis admitted to the intensive care unit (ICU) of the First Affiliated Hospital of China Medical University between April 20, 2017, and December 31, 2024, constituted the development cohort. Latent class trajectory modeling (LCTM) was applied to classify patients into different subclasses based on the AT Ⅲ level trajectories over the first 7 days after sepsis diagnosis. Clinical characteristics and outcomes were compared among these subclasses. Additionally, the AT Ⅲ trajectory patterns were validated in an external cohort of IAI patients derived from the China Multicenter Sepsis dataset.

**Results:**

Four dynamic AT Ⅲ trajectory subclasses were identified and further validated by data from the development cohort (*n*=779) and the external validation cohort (*n*=820): Class 1 exhibited initially low AT Ⅲ levels with a rapid decline during the first 3 days; Class 2 showed initially low AT Ⅲ followed by gradual recovery; Class 3 started with normal AT Ⅲ levels but experienced a sharp decline in the first 3 days; Class 4 maintained AT Ⅲ levels within the normal range throughout. In the development cohort, patients in Class 1 demonstrated the most pronounced coagulation deterioration, characterized by the lowest platelet counts, significantly prolonged prothrombin time (PT) and activated partial thromboplastin time (APTT), more severe inflammatory response, and elevated lactate levels, with the highest ICU and 30-day mortality. Moreover, Class 1 consistently showed significantly higher SOFA scores within 7 days after sepsis diagnosis compared to other subgroups. Incorporating the identification of Class 1 dynamic trajectory significantly improved the predictive performance for 30-day mortality (area under the curve = 0.824, *P* = 0.0015).

**Conclusions:**

This prospective cohort study uncovers heterogeneity in AT Ⅲ trajectories among IAI-induced sepsis, which were closely associated with the disease severity over time. Incorporating Class 1 (initial low AT followed by rapid decline) improves the predictive value for sepsis prognosis.

## Introduction

Acute intra-abdominal infections (IAIs) are a common spectrum of severe surgical emergencies. IAI represented 22%–35% of the cause of sepsis or septic shock.^[^^1^^,^^2^^]^ Approximately 30% of patients admitted to the ICU with IAI succumb to the disease.^[^^3^^]^ In the IAI-induced sepsis, the crosstalk between excessive inflammation and coagulation disorders underlies the onset of multiple organ dysfunction.^[^^4^^,^^5^^]^ While the inflammatory response is well-recognized by surgeons, the coagulation disorders induced by IAI have received considerably less attention. With the growing recognition of sepsis-induced coagulopathy, it is considered a complex syndrome characterized by over-activation of the coagulation system and suppression of fibrinolytic pathways, resulting in intravascular clotting formation, endothelial injury, and even multiorgan dysfunction.^[^^6^^]^

Antithrombin III (AT Ⅲ), the most abundant and essential endogenous anticoagulant in humans, plays a crucial role in regulating coagulation and fibrinolysis.^[^^7^^]^ It is mainly synthesized by the liver and is an essential inhibitor of thrombin and factor Xa. Its anticoagulatory activity can be enhanced by heparin.^[^^7^^]^ In addition to its anticoagulation function, AT Ⅲ induces anti-inflammatory signaling by binding to glycosaminoglycans on vascular endothelial cells and promoting the phosphorylation of syndecan-4.^[^^8^^]^ Moreover, AT Ⅲ also exhibits antimicrobial activity, as peptide fragments derived from its proteolytic degradation possess antimicrobial properties.^[^^9^^]^ During sepsis, AT Ⅲ levels are significantly reduced due to increased consumption resulting from endothelial injury and coagulation activation, impaired hepatic synthesis, and degradation of AT Ⅲ by neutrophil elastase.^[^^10^^]^ Emerging Studies have demonstrated that the levels of AT Ⅲ are significantly decreased among septic patients.^[^^11^^]^ In patients with severe abdominal infections, AT Ⅲ levels are significantly lower in non-survivors compared to survivors.^[^^12^^]^ AT Ⅲ level remains around 80% in septic patients without organ dysfunction, whereas in those with organ dysfunction, AT Ⅲ level decreases to approximately 60%.^[^^13^^]^ Studies have also demonstrated that low plasma AT Ⅲ levels in sepsis patients are significantly associated with increased mortality and serve as an independent predictor of clinical outcomes.^[^^14^^,^^15^^]^

Currently, most clinical studies on AT Ⅲ and sepsis are based on single time-point measurements, typically assessing baseline levels at the time of sepsis diagnosis. Due to the high heterogeneity of sepsis, the severity of endothelial injury and liver dysfunction varies greatly among patients, contributing to the wide variation in AT Ⅲ levels at diagnosis.^[^^16^^]^ For IAI-induced sepsis that is predominantly caused by Gram-negative bacilli, the association between the dynamic changes in AT Ⅲ and the disease progression remains unclear. Therefore, we applied latent class trajectory modeling (LCTM) to classify IAI-induced sepsis patients into four distinct subgroups based on their AT Ⅲ trajectory over the first 7 days, and validated the LCTM model in the China Multicenter Sepsis database. Then we evaluated the association between AT Ⅲ trajectories and disease progression in IAI-induced sepsis.

## Methods

### Study cohort

This was a prospective observational study. The development cohort enrolled patients with sepsis caused by IAI admitted to the intensive care unit (ICU) of the First Hospital of China Medical University (CMU-ICU) between April 20, 2017, and December 31, 2024. Two reviewers independently reviewed patients’ clinical records and assigned the etiology of sepsis to ensure the accuracy and reliability of data. The validation cohort was extracted from the China Multicenter Sepsis (CMS) dataset with clinical records of adult sepsis patients from January 1, 2023 to December 31, 2024, involving 18 ICUs from tertiary university hospitals across diverse geographic regions in China. Inclusion criteria: patients diagnosed with IAI-induced sepsis. Sepsis was defined according to the Sepsis 3.0 criteria: patients had a documented or suspected infection along with an acute increase of ≥2 points in the Sequential Organ Failure Assessment (SOFA) score. IAI was defined as an infection originating from the abdominal cavity, involving the digestive organs and the peritoneum, and encompasses a spectrum of conditions ranging from appendicitis to peritonitis. Exclusion criteria: (1) pregnant, (2) age<18 years, (3) <48 h stayed in ICU, (4) absence of follow-up records, (5) preexisting severe liver diseases (Child-Pugh B or more), or (6) major bleeding at enrollment. To ensure the accuracy of the latent class model based on longitudinal data over time, patients need to have four measurements within 7 days upon enrollment. Therefore, patients with fewer than four measurements of AT Ⅲ and those without abdominal infections were excluded. The study was approved by the Research and Ethics Committee of the First Affiliated Hospital of China Medical University ([2022] 2022-502-2, Shenyang, China). The other participating ICUs obtained their respective ethical approvals on CMS data. Written informed consent was obtained from each participant or their legal surrogates before enrollment.

### Data collection and outcomes

The baseline information, including demographic and clinical data, was obtained from the hospital’s electronic medical record system. Demographic data included age, sex, body mass index (BMI), and comorbidities. Clinical scores, such as the Acute Physiology and Chronic Health Evaluation II (APACHE II) score, the International Society on Thrombosis and Haemostasis (ISTH) score, and the Japanese Association for Acute Medicine (JAAM) score, were calculated based on the worst values within 24 h of sepsis diagnosis. The first measurement of laboratory variables upon enrollment, including white blood cell counts (WBC), lymphocyte counts (LC), procalcitonin (PCT), C-reactive protein (CRP), Interleukin-6 (IL-6), platelet counts (PLT), prothrombin time (PT), activated partial thromboplastin time (APTT), fibrinogen (Fg), D-dimer (DD), fibrin degradation products (FDP), lactic acid (Lac), creatinine (Cr), and total bilirubin (TBIL) were extracted in both cohorts. In addition, the development cohort also collected some nutritional, endocrine, and immune markers, such as hemoglobin (Hb), total protein (TP), albumin (Alb), free triiodothyronine (FT3), free thyroxine (FT4), and CD3^+^, CD4^+^, and CD8^+^T cell counts. AT Ⅲ level was collected continuously for the first 7 days following the diagnosis of sepsis, along with the SOFA score. An AT Ⅲ level above 70% was considered within the normal range. The outcomes included ICU mortality, 30-day mortality, length of ICU stay, duration of mechanical ventilation, incidence of septic shock, incidence of disseminated intravascular coagulation (DIC), and use of continuous renal replacement therapy (CRRT). ICU mortality is defined as the death of a patient occurring during their stay in the ICU, irrespective of the underlying cause. Length of ICU stay refers to the number of days stayed in the ICU. 30-Day mortality refers to the death of a patient within 30 days from the beginning of sepsis. DIC was in accordance with the ISTH or JAAM criteria. Septic shock is defined as persistent hypotension requiring vasopressors to maintain mean arterial pressure (MAP) ≥65 mmHg despite adequate fluid resuscitation, along with a serum lactate level >2 mmol/L (18 mg/dL).

### Statistical analysis

Descriptive results for the characteristics of different subclasses are presented as either the mean ± standard deviation (SD) or the median with interquartile range (IQR), depending on whether the data conform to a normal distribution. Comparisons of continuous variables between two groups were performed with the Student’s *t*-test when the assumption of normality was met (assessed by the Shapiro–Wilk test). Otherwise, the nonparametric Mann–Whitney *U* test was applied. Continuous variables were compared across trajectories using one-way ANOVA followed by Dunnett’s multiple comparisons test when assumptions of normality and homogeneity of variance were met; otherwise, the Kruskal–Wallis test was applied. Categorical variables were compared using the Chi-squared test.

LCTM was conducted to classify the dynamic trajectories of AT Ⅲ in patients with IAI-induced sepsis. The optimal number of classes was determined using the Akaike information criterion (AIC), Bayesian information criterion (BIC), entropy, and proper clinical interpretability. The final model of four subclasses was determined by lower AIC and BIC values, higher entropy, and a minimum subclass size of 2% of the total cohort, along with the average posterior probability of group membership ≥70% for each class (Supplementary Table S1). The fixed effect coefficients based on maximum likelihood estimation were presented in the Supplementary Table S2. The final model was applied to the validation cohort, in which individual AT Ⅲ data from each patient were fitted to the mixture polynomial components of the trained LCTM. The mean squared errors (MSE) were calculated for each component’s fit. Patients were then assigned to the subclass corresponding to the component that yielded the lowest MSE.

We used logistic regression analysis to evaluate the relationship between the classification of sepsis patients based on the AT Ⅲ trajectories and the clinical outcomes. To assess the independent impact of the classification of included patients based on AT Ⅲ trajectory on these outcomes, the logistic regression analysis was performed in an unadjusted model and adjusted for age, sex, and SOFA score. The evaluation indicators were odds ratios (ORs) and 95% confidence intervals (CIs). The Kaplan–Meier survival curve was used to assess the relationship between patients with sepsis based on the AT Ⅲ trajectory and 30-day mortality. We plotted the receiver operating characteristic curve (ROC) for the combination of SOFA score, IL-6, PCT, PLT, and the AT Ⅲ dynamic trajectory to predict the 30-day mortality of sepsis patients. We conducted a mediation analysis to examine both direct and indirect effects, with the magnitude and significance of the mediation effects assessed using nonparametric bootstrapping with 1000 resamples. The models were adjusted for baseline variables that differed significantly across subclasses. All data were analyzed using Python (version 3.10, Wilmington, DE, USA) and R (version 4.2.3, Vienna, Austria) statistical software.

## Results

**Figure 1:**
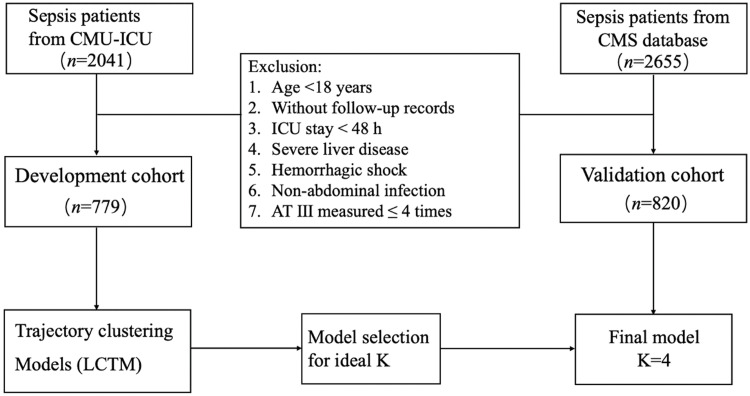
Flow chart of the development cohort and validation cohort. LCTM: Latent class trajectory modeling.

A total of 2041 sepsis patients were initially identified from the CMU-ICU dataset. After applying the exclusion criteria, 779 patients with IAI were ultimately included in the development cohort. A total of 2655 patients were diagnosed with sepsis in the CMS dataset, and 820 patients with IAI were finally included in the validation cohort (Figure 1). The development cohort included 67.8% male, with a median age of 67.0 years, a median SOFA of 8.0, and an overall ICU mortality of 21.0%, and 30-day mortality of 27.1% (Table 1). In the validation cohort, 60.1% patients were male, with a median age of 67.0 years. The median SOFA score was 9.0, and ICU and 30-day mortality were 11.2% and 13.8%, respectively (Supplementary Table S3).

**Table 1:** Clinical characteristics among the four classes of IAI-induced sepsis in the development cohort.

Characteristics	Overall (*n*=779)	Class 1 (*n*=197)	Class 2 (*n*=320)	Class 3 (*n*=111)	Class 4 (*n*=151)	*P* value
Age (years)	67.0 (57.0–76.0)	67.0 (58.0–75.0)	67.0 (58.0–75.0)	69.0 (59.0–79.5)	62.0 (52.0–75.0)	0.004
Male	528 (67.8)	116 (58.9)	223 (69.7)	78 (70.3)	111 (73.5)	0.016
BMI (kg/m²)	23.7 (21.3–26.1)	22.9 (20.4–25.3)	23.9 (21.2–26.1)	23.4 (21.2–26.9)	24.5 (22.6–26.3)	<0.001
Comorbidities						
Hypertension	258 (33.1)	54 (27.4)	105 (32.8)	44 (39.6)	55 (36.4)	0.122
DM	139 (17.8)	25 (12.7)	58 (18.1)	19 (17.1)	37 (24.5)	0.042
Malignancy	122 (15.7)	45 (22.8)	44 (13.8)	18 (16.2)	15 (9.9)	0.006
COPD	14 (1.8)	4 (2.0)	4 (1.3)	2 (1.8)	4 (2.6)	0.671
Clinical scores						
APACHE II score	16.0 (12.0–19.0)	16.0 (14.0–20.0)	15.0 (11.8–18.0)	17.0 (14.0–20.5)	14.0 (11.0–20.0)	<0.001
SOFA score	8.0 (5.0–10.0)	8.0 (6.0–11.0)	7.0 (5.0–10.0)	7.0 (5.0–10.0)	7.0 (5.0–9.0)	<0.001
ISTH DIC score	3.0 (2.0–4.0)	4.0 (2.0–5.0)	2.5 (2.0–4.0)	3.0 (2.0–3.0)	3.0 (2.0–3.0)	<0.001
JAAM DIC score	3.0 (1.0–4.0)	3.0 (2.0–5.0)	2.0 (1.0–4.0)	2.0 (1.0–4.0)	2.0 (1.0–4.0)	<0.001
Outcomes						
DIC	214 (27.5)	115 (58.4)	58 (18.1)	27 (24.3)	14 (9.3)	<0.001
Septic shock	558 (71.6)	171 (86.8)	225 (70.3)	83 (74.8)	79 (52.3)	<0.001
CRRT	153 (19.6)	70 (35.5)	42 (13.1)	22 (19.8)	19 (12.6)	<0.001
MV duration (hours)	83 (43–175)	134 (80–222)	64 (40–140)	92 (51–191)	67 (37–144)	<0.001
ICU LOS (days)	6.0 (4.0–11.0)	7.0 (5.0–14.0)	5.0 (4.0–9.0)	6.0 (4.0–11.0)	5.0 (3.0–9.5)	<0.001
ICU mortality	164 (21.0)	68 (34.5)	42 (13.1)	30 (27.0)	24 (15.9)	<0.001
30-Day mortality	211 (27.1)	82 (41.6)	64 (20.0)	31 (27.9)	34 (22.5)	<0.001

Data are presented as median (interquartile range) for continuous variables and *n* (%) for categorical variables.

APACHE II: Acute Physiology and Chronic Health Evaluation II; BMI: Body mass index; COPD: Chronic obstructive pulmonary disease; CRRT: Continuous renal replacement therapy; DIC: Disseminated intravascular coagulation; DM: Diabetes mellitus; IAI: Intra-abdominal infection; ICU: Intensive care unit; JAAM: Japanese Association for Acute Medicine; LOS: Length of stay; MV: Mechanical ventilation; SOFA: Sequential Organ Failure Assessment.

### Clinical characteristics of AT Ⅲ trajectory subclasses

**Figure 2:**
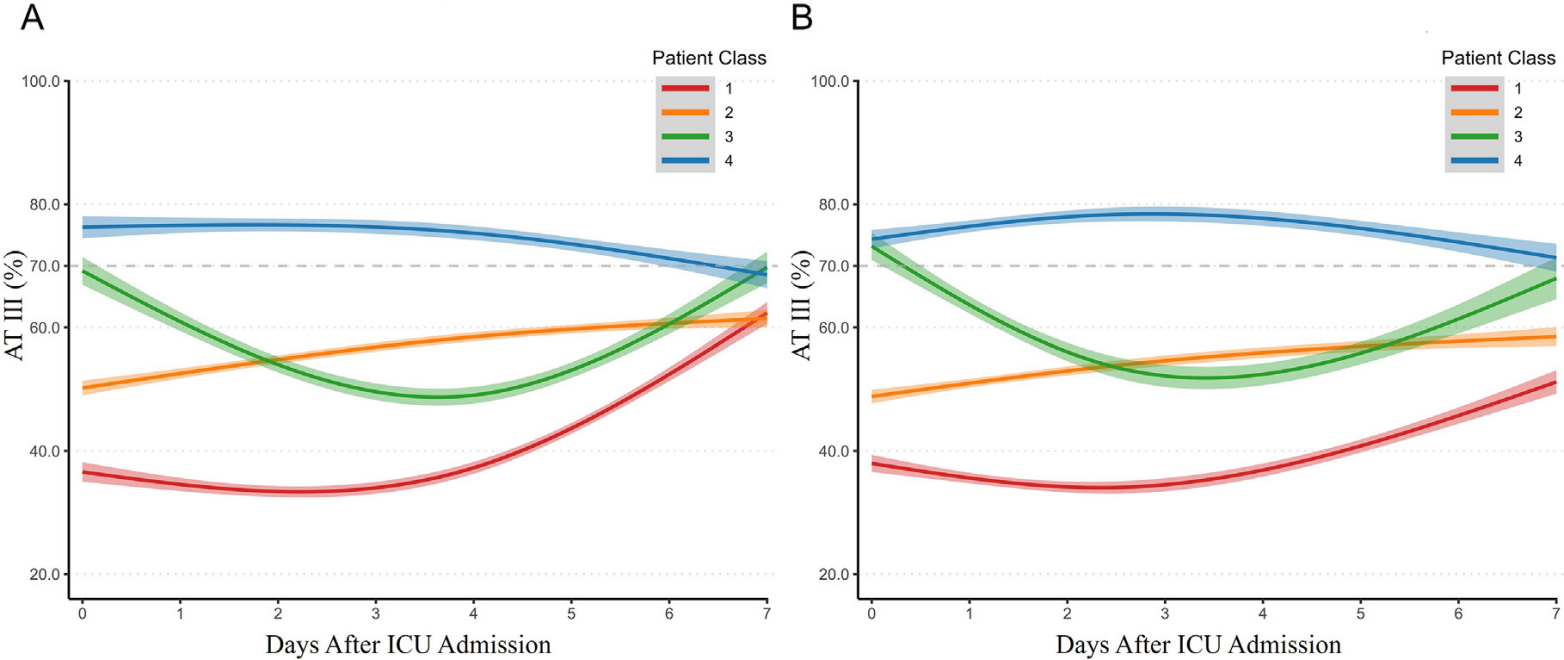
Latent class trajectory modeling of AT Ⅲ during the first 7 days of sepsis. A: The AT Ⅲ trajectories in the development cohort. Class 1 (*n*=197, 25.3%) represents a low AT Ⅲ followed by a rapid decline within the first 3 days; Class 2 (*n*=320, 41.1%) shows an initial low AT Ⅲ with a gradual increase; Class 3 (*n*=111, 14.2%) has a baseline normal AT Ⅲ followed by a sharp decrease; Class 4 (*n*=151, 19.4%) shows a stable and normal AT Ⅲ levels. B: The AT Ⅲ trajectories in the validation cohort. Class 1 (*n*=193, 23.5%), Class 2 (*n*=310, 37.8%), Class 3 (*n*=92, 11.2%), Class 4 (*n*=225, 27.4%). AT Ⅲ ≥70% was defined as within the normal range. The shaded area of the curve represents the 95% confidence intervals of each trajectory. AT Ⅲ: Antithrombin Ⅲ; ICU: Intensive care unit.

The development cohort was classified into four AT Ⅲ trajectory subclasses (Figure 1A): Class 1 (*n*=197, 25.3%) represents a low AT Ⅲ followed by a rapid decline within the first 3 days; Class 2 (*n*=320, 41.1%) shows an initial low AT Ⅲ with a gradually increase; Class 3 (*n*=111, 14.2%) has a baseline normal AT Ⅲ followed by a sharp decrease; Class 4 (*n*=151, 19.4%) shows a stable and normal AT Ⅲ levels. Compared with other classes, Class 1 had the highest prevalence of history of malignancy (22.8%), the lowest median BMI (22.9 kg/m²), and the highest SOFA score of 8 and DIC scores (ISTH score: 4, JAAM score: 3). In terms of clinical outcomes, Class 1 exhibited higher incidences of DIC (58.4%), septic shock (86.8%), and longer ICU stay (7 days), required more organ support (including more frequent use of CRRT and longer mechanical ventilation), and had the highest ICU mortality (34.5%, *P* < 0.001) and 30-day mortality (41.6%, *P* < 0.001) (Table 1 and Figure 2A). In the external validation cohort, four trajectory classes similar to those identified in the development cohort were derived (Figure 2B): Class 1 (*n*=193, 23.5%), Class 2 (*n*=310, 37.8%), Class 3 (*n*=92, 11.2%), and Class 4 (*n*=225, 27.4%). Consistent with the development cohort, Class 1 exhibited the highest disease severity and had higher ICU (19.2%) and 30-day mortality rates (19.7%) compared to the other three classes (*P*<0.05, Supplementary Table 3). No significant differences were observed among the four classes in terms of age, sex, CRRT, MV duration, or length of ICU stay.

### Laboratory parameters comparison among four AT Ⅲ trajectory subclasses

**Figure 3:**
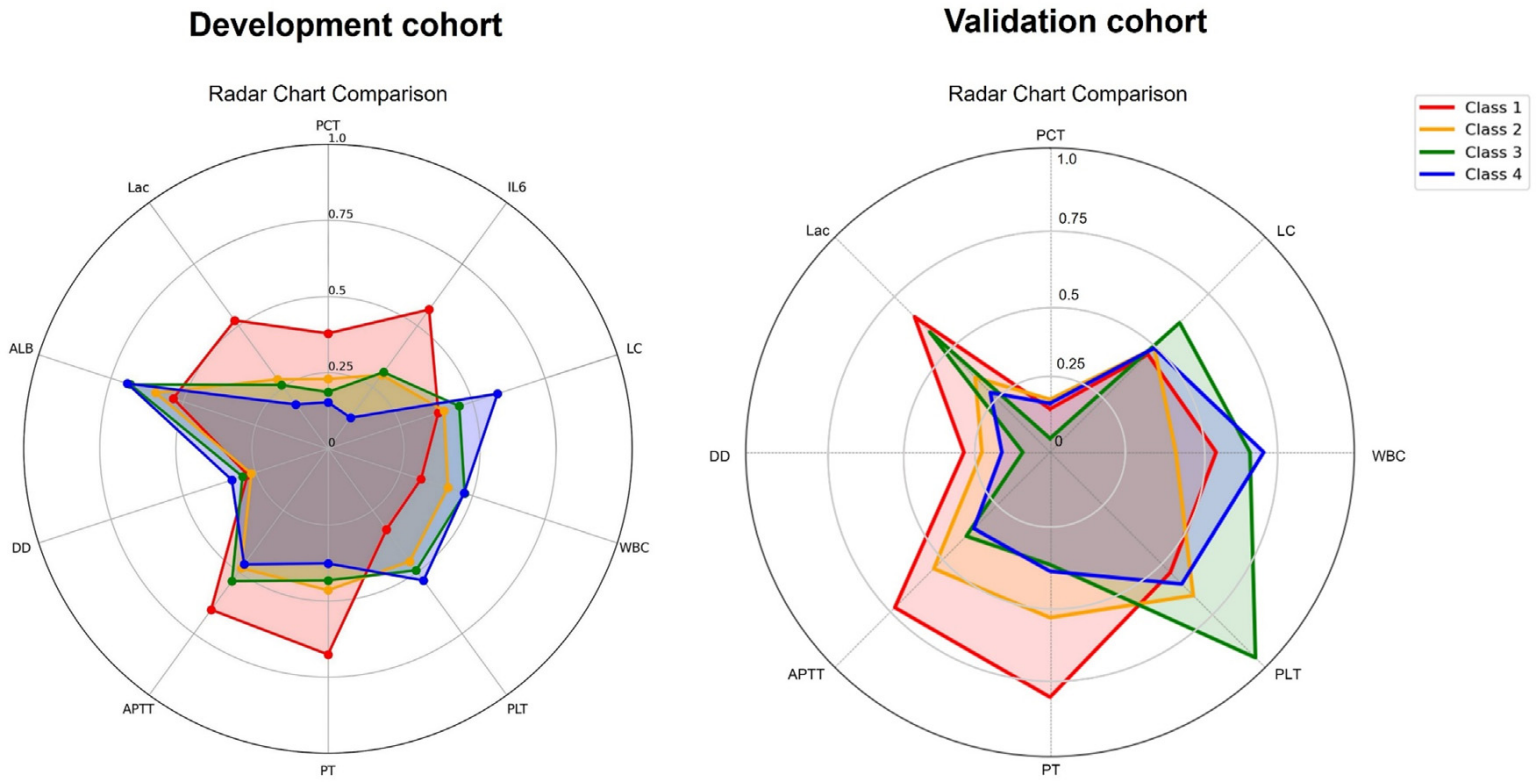
Radar charts of both cohorts. Radar chart shows the laboratory parameters with statistically significant differences among the four classes in the development cohort. ALB: Albumin; APTT: Activated partial thromboplastin time; DD: D-dimer; Lac: Lactate; IL-6: Interleukin-6; LC: Lymphocyte count; PCT: Procalcitonin; PLT: Platelet count; PT: Prothrombin time; WBC: White blood cell count.

In the development cohort, Class 1 exhibited a markedly heightened inflammatory response, characterized by significantly elevated levels of PCT and IL-6 compared to the other subclasses (Class 1: PCT 18 ng/mL, IL-6 1422 pg/mL, *P* < 0.001). In addition, Class 1 showed pronounced coagulation activation, with significantly prolonged PT and APTT, and notably reduced platelet counts (Class 1: PT 18.8 s, APTT 54.1 s, PLT 114 × 10^9^/L, *P* < 0.001). Concurrently, fibrinolysis appeared suppressed, as indicated by significantly lower levels of fibrinogen and D-dimer. Additionally, Class 1 had the lowest white blood cell counts (7.2 × 10⁹/L), lymphocyte counts (0.6 × 10⁹/L), and T-cell counts, as well as the highest lactate concentration (3.5 mmol/L, *P* < 0.001) (Table 2). The radar chart clearly illustrated significant differences in laboratory parameters among the four classes (*P* < 0.01, Figure 3A). Consistent with the development cohort, Class 1 also exhibited obvious coagulation disorder, characterized by prolonged PT and APTT, elevated D-dimer, and reduced platelet counts in the validation cohort (Figure 3B). In summary, Class 1 was characterized by heightened inflammatory and coagulation responses, markedly reduced immune cell counts, and a greater need for organ support, suggesting a potential association with poor sepsis outcomes.

**Table 2:** The laboratory variables across the four subclasses in the development cohort.

Variables	Overall (*n*=779)	Class 1 (*n*=197)	Class 2 (*n*=320)	Class 3 (*n*=111)	Class 4 (*n*=151)	*P*value
CRP (mg/L)	181.5 (104.1-244.8)	171.8 (98.0-235.8)	185.2 (114.1-248.4)	163.0 (98.3-234.8)	197.6 (103.3-260.8)	0.209
PCT (ng/mL)	9.2 (2.0-31.6)	18.0 (4.8-80.1)	9.5 (2.1-25.9)	8.1 (2.9-18.9)	3.5 (0.8-15.5)	<0.001
Hb (g/L)	99.0 (82.0-119.5)	91.0 (79.0-113.0)	103.0 (85.8-123.0)	96.0 (77.0-117.5)	105.0 (83.0-121.5)	<0.001
PLT (× 10⁹/L)	153 (104-220)	114 (77-166)	162 (110-231)	171 (113-245)	185 (135-266)	<0.001
LC (× 10⁹/L)	0.7 (0.5-1.1)	0.6 (0.4-0.9)	0.7 (0.5-1.1)	0.8 (0.5-1.1)	0.9 (0.6-1.3)	<0.001
ALB (g/L)	23.9 (19.6-27.4)	22.4 (16.0-26.6)	22.9 (19.1-26.2)	25.7 (21.6-29.1)	25.5 (22.9-28.9)	<0.001
TP (g/L)	45.8 (38.9-51.1)	40.9 (33.3-46.6)	44.3 (38.2-49.1)	49.3 (45.8-52.8)	50.3 (45.8-55.2)	<0.001
CD4⁺ (cells/µL)	247 (142-399)	184 (103-321)	241 (143-374)	291 (168-454)	320 (208-474)	<0.001
CD8⁺ (cells/µL)	136 (74-240)	118 (56-183)	124 (59-213)	168 (92-254)	184 (109-334)	<0.001
CD3⁺ (cells/µL)	416 (239-660)	314 (221-520)	399 (214-630)	466 (268-732)	516 (332-861)	<0.001
FT3 (pg/mL)	1.8 (1.5-2.2)	1.6 (1.5-2.0)	1.8 (1.5-2.2)	1.8 (1.5-2.3)	1.9 (1.6-2.3)	0.001
FT4 (pmol/L)	11.9 (9.6,--14.2)	11.3 (9.1-13.2)	12.2 (10.1-14.5)	11.9 (9.7-14.6)	12.2 (10.1-14.4)	0.002
PT (s)	16.6 (15.0-18.8)	18.8 (16.7-22.1)	16.4 (15.0-18.3)	16.0 (14.4-17.3)	15.4 (14.3-16.7)	<0.001
APTT (s)	45.9 (38.7-55.3)	54.1 (45.3-66.6)	44.5 (38.2-52.0)	43.2 (35.8-53.9)	42.2 (36.9-50.5)	<0.001
Fb (g/L)	4.4 (3.0-5.8)	3.2 (2.4-4.7)	4.5 (3.2-5.7)	4.6 (3.5-5.6)	5.4 (4.1-7.5)	<0.001
DD (mg/L)	3.8 (2.6-6.7)	3.4 (2.3-6.1)	3.7 (2.7-6.2)	4.4 (2.6-7.5)	4.5 (2.8-8.4)	0.014
FDP (mg/L)	14.6 (8.7-27.2)	12.3 (8.2-24.3)	15.1 (8.9-25.5)	15.4 (9.1-28.9)	17.1 (9.9-33.5)	0.089
WBC (× 10⁹/L)	9.9 (4.9-16.0)	7.2 (3.2-12.6)	9.9 (4.8-16.0)	10.8 (6.7-17.9)	12.1 (8.0-17.2)	<0.001
IL-6 (pg/mL)	446 (78-2500)	1422 (97-5000)	500 (82-2500)	403 (74-2500)	138 (66-729)	<0.001
Lac (mmol/L)	2.0 (1.2-3.5)	3.5 (1.9-5.3)	1.9 (1.2-3.0)	1.8 (1.1-3.0)	1.4 (1.1-2.0)	<0.001
Cr (µmol/L)	92.0 (62.5-162.5)	103.0 (67.0-171.0)	89.0 (62.0-137.0)	91.0 (62.0-146.0)	88.0 (60.5-197.5)	0.190
TBil (µmol/L)	21.4 (13.8-35.8)	20.7 (13.5-35.9)	22.0 (14.0-36.8)	22.3 (14.3-35.0)	20.4 (13.6-32.0)	0.779

Data are presented as median (interquartile range) for all laboratory variables.

APTT: Activated partial thromboplastin time; ALB: Albumin; Cr: Creatinine; CD8⁺: CD8-positive T-lymphocytes; CD3⁺: CD3-positive T-lymphocytes; CD4⁺: CD4-positive T-lymphocytes; CRP: C-reactive protein; DD: D-dimer; FT3: Free triiodothyronine; FT4: Free thyroxine; Fb: Fibrinogen; FDP: Fibrinogen degradation products; Hb: Hemoglobin; IL-6: Interleukin-6; LC: Lymphocyte count; Lac: lactate; PLT: Platelet count; PCT: Procalcitonin; PT: Prothrombin time; TP: Total protein; TBil: Total bilirubin; WBC: White blood cell count.

Analysis of the validation cohort showed that Class 1 exhibited more pronounced coagulation dysfunction, characterized by prolonged PT and APTT, lower fibrinogen levels, and the highest lactate concentration. However, no significant differences in inflammatory markers or immune cell counts were observed among the four subclasses (Supplementary Table S4).

### Association of subclasses with mortality as well as the severity of organ dysfunction

**Figure 4:**
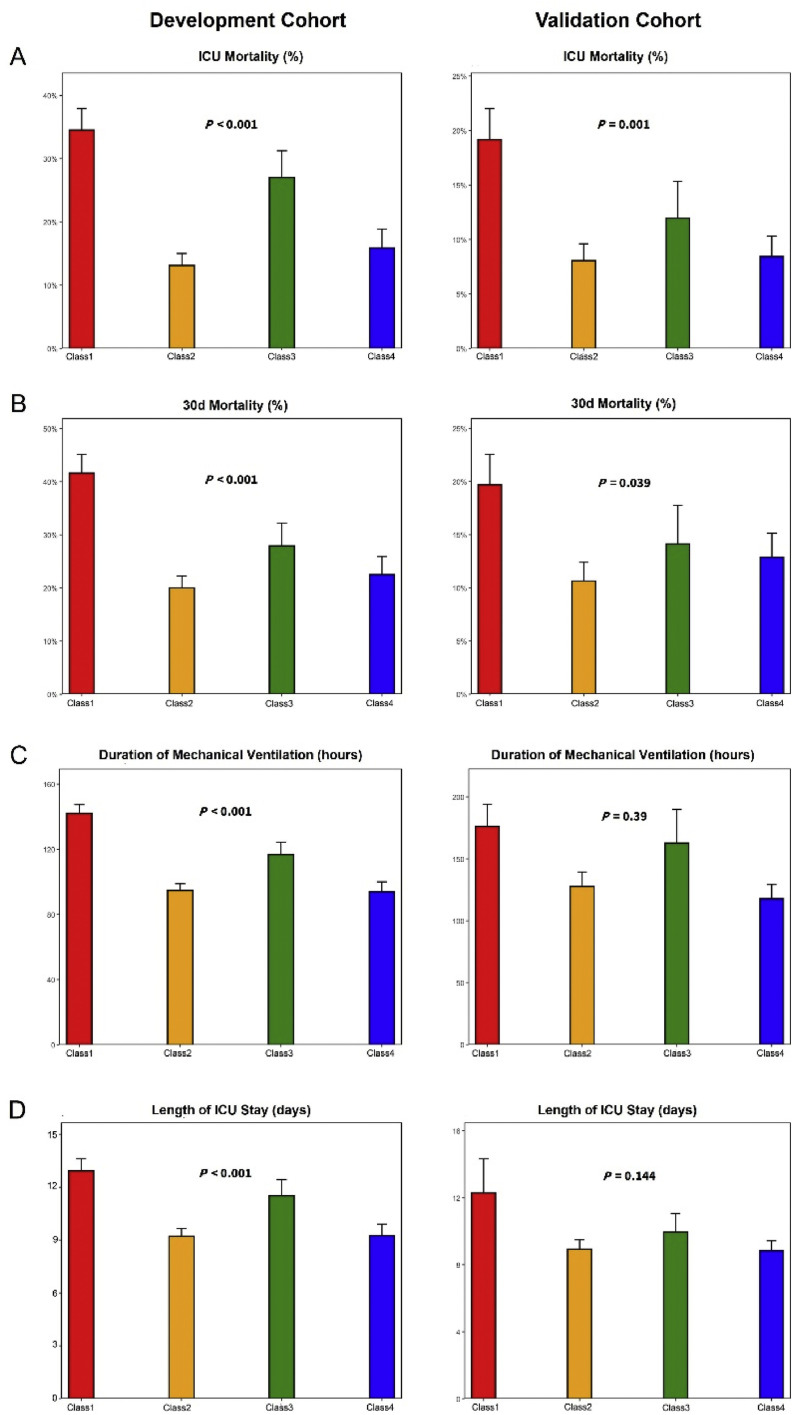
The outcomes including ICU mortality(A), 30-day mortality (B), mechanical ventilation duration (C), and the length of ICU stay (D) show statistically significant differences across the four classes in the development and validation cohorts.The laboratory parameter levels represent the mean and standard error of the mean (SEM). ICU: Intensive care unit.

**Figure 5:**
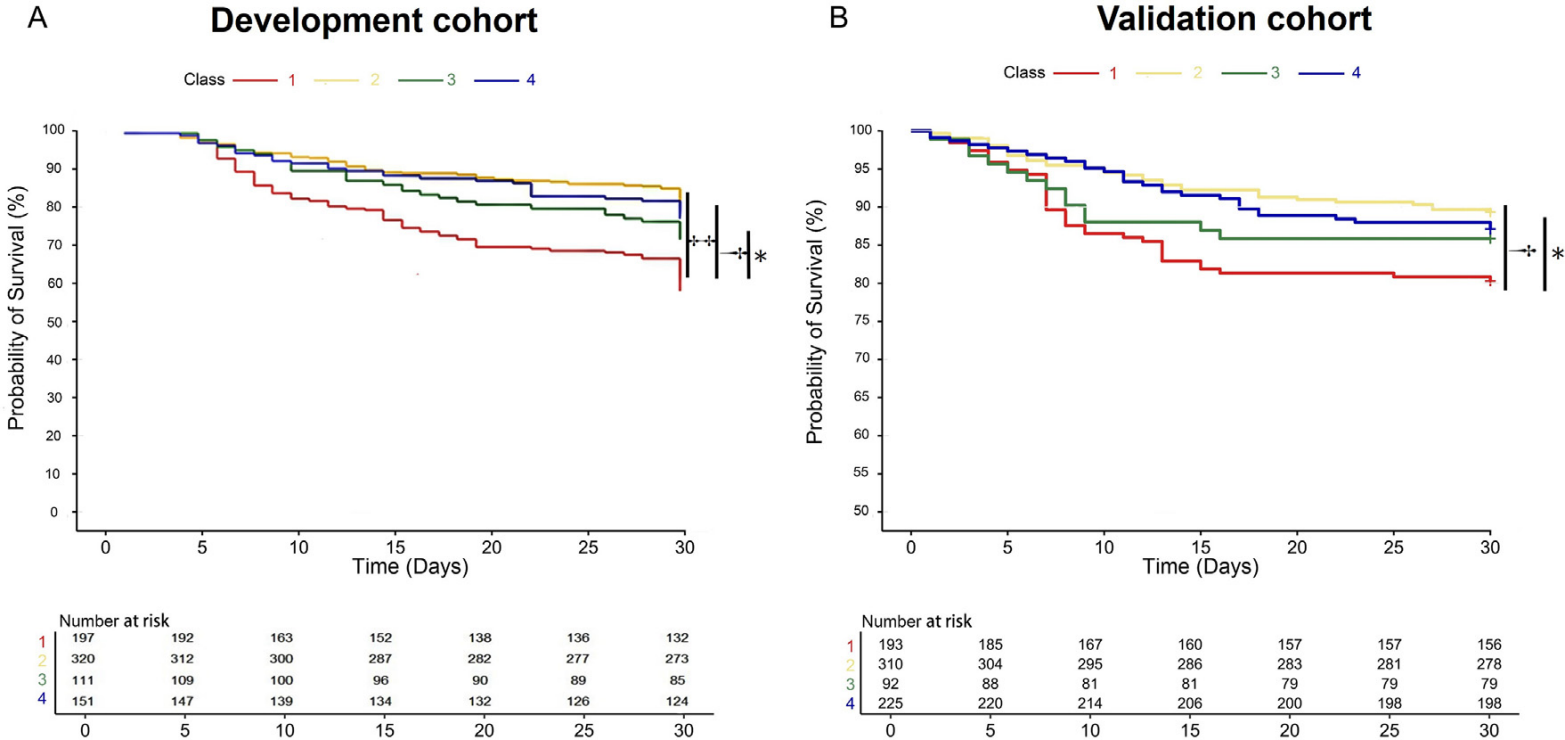
Kaplan–Meier survival curves of the four trajectories in the development (A) and validation cohorts (B). ^⁎^*P* < 0.05, ^†^*P* < 0.001, ^‡^*P* < 0.0001.

The comparison of clinical outcomes among the four subgroups in the development cohort and validation cohort showed that Class 1 had the highest ICU and 30-day mortality rates, and the longest duration of mechanical ventilation and ICU stay (*P* <0.001, Figure 4). To further investigate the relationship between Class 1 and clinical outcomes, we used the worst prognosis group, Class 1, as the reference. After adjusting for confounding factors (age, sex, BMI, DM, Malignancy, SOFA and APACHE II score), multivariable logistic regression analysis revealed that Class 2 and Class 4 had a lower risk of ICU mortality (Class 2: OR=0.33, 95% CI: 0.21 to 0.52, *P* <0.001, Class 4: OR=0.42, 95% CI: 0.24 to 0.73, *P*=0.003). Regardless of whether confounding factors were adjusted for, Class 1 patients consistently exhibited the highest 30-day mortality rate (Table 3). Kaplan–Meier survival analysis further confirmed that Class 1 had the highest 30-day mortality rate, followed by Class 3, Class 4, and the lowest rate in Class 2 in both cohorts (Figure 5).

**Table 3:** Association of subclasses with ICU and 30-day mortality in the development cohort.

AT Ⅲ trajectories	ICU mortality		30-Day mortality	
	OR (95% CI)	*P* value	OR (95% CI)	*P*-value
Unadjusted				
Class 1	1.00 (ref.)		1.00 (ref.)	
Class 2	0.29 (0.19–0.44)	<0.001	0.35 (0.24–0.52)	<0.001
Class 3	0.70 (0.42–1.17)	0.176	0.54 (0.33–0.90)	0.017
Class 4	0.36 (0.21–0.61)	<0.001	0.41 (0.25–0.66)	<0.001
Adjusted*				
Class 1	1.00 (ref.)		1.00 (ref.)	
Class 2	0.31 (0.20–0.48)	<0.001	0.36 (0.24–0.53)	<0.001
Class 3	0.75 (0.44–1.26)	0.277	0.54 (0.32–0.90)	0.017
Class 4	0.43 (0.25–0.73)	0.002	0.43 (0.26–0.70)	<0.001
Adjusted†				
Class 1	1.00 (ref.)		1.00 (ref.)	
Class 2	0.33 (0.21–0.52)	<0.001	0.38 (0.25–0.58)	<0.001
Class 3	0.70 (0.41–1.20)	0.199	0.52 (0.31–0.88)	0.015
Class 4	0.42 (0.24–0.73)	0.003	0.44 (0.26–0.73)	0.002

APACHE II: Acute Physiology and Chronic Health Evaluation II; BMI: Body mass index; DM: Diabetes mellitus; ICU: Intensive care unit; SOFA: Sequential Organ Failure Assessment.

⁎ Adjusted for age, sex, and SOFA score.

† Adjusted for age, sex, BMI, DM, malignancy, SOFA score, and APACHE II score.

**Figure 6:**
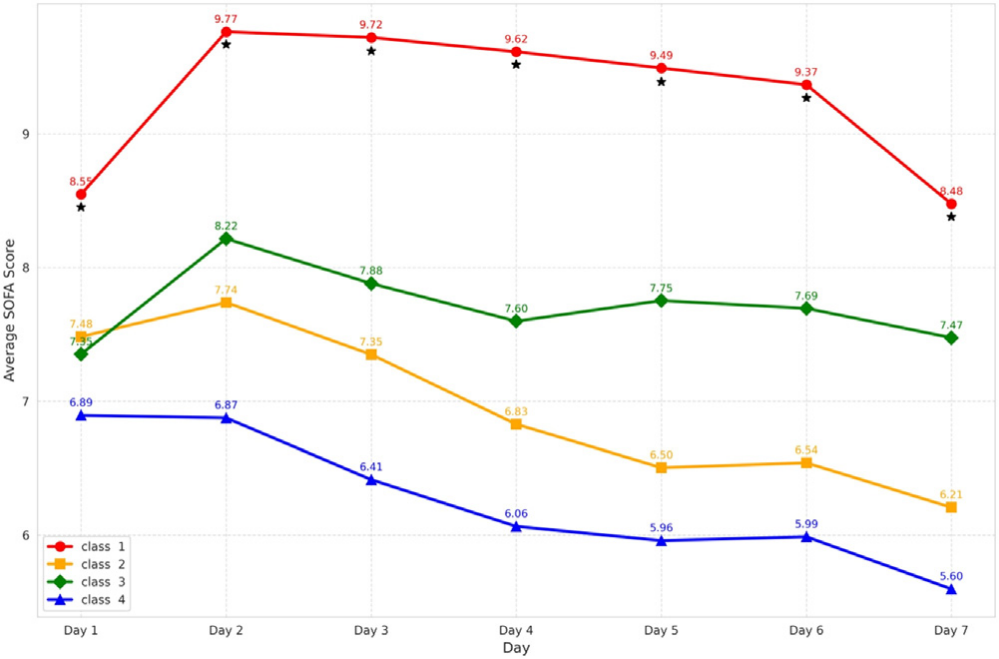
The dynamic changes in SOFA scores across the four classes in the first 7 days of sepsis in the development cohort. ^⁎^*P* < 0.05 shows the comparison of mean values of SOFA scores using the Kruskal–Wallis test at each time point. SOFA: Sequential Organ Failure Assessment.

**Figure 7:**
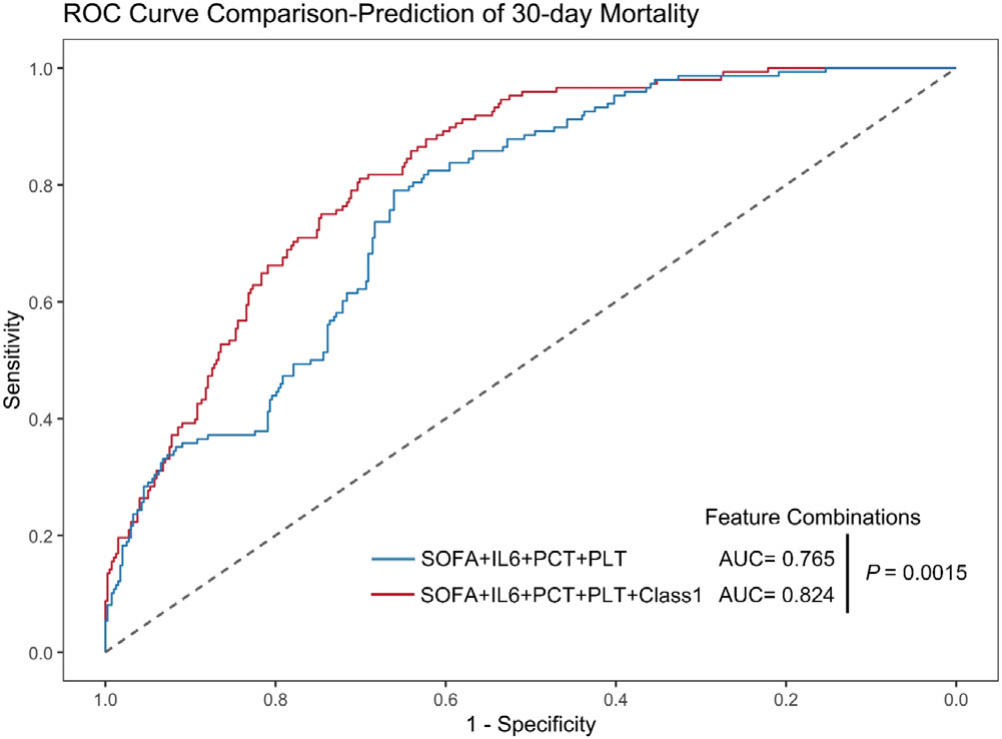
Comparison of ROC curves for 30-day mortality prediction. The blue curve represents the predictive performance of the combination of SOFA score, IL-6, PCT, and PLT, while the red curve represents the predictive performance of the SOFA score, IL-6, PCT, and PLT combination with Class 1 (low AT, rapid decline). The Delong test was used to compare the AUC of the two curves, showing a significant improvement in predictive performance for the combination of SOFA and Class 1 (*P* = 0.0015). AT: Antithrombin; IL-6: Interleukin-6; PCT: Procalcitonin; PLT: Platelet count; ROC: Receiver operating characteristic; SOFA: Sequential Organ Failure Assessment.

Given the close association between dynamic changes in AT Ⅲ levels and inflammatory responses and coagulation dysfunction, we further investigated the relationship between AT Ⅲ trajectories and organ dysfunction. We tracked the dynamic changes in SOFA scores over the first 7 days after ICU admission among the four subgroups in the development cohort. The results showed that Class 1 not only had the highest initial SOFA score but also demonstrated persistent organ dysfunction, with significantly higher scores than the other subgroups through Day 7 (Figure 6). Based on this persistent organ dysfunction pattern, along with higher inflammatory response and worse coagulation index in Class 1, we hypothesized that incorporating this phenotype could enhance the predictive value for the prognosis of sepsis. Our result confirmed that the inclusion of Class 1 phenotype, based on SOFA score, IL-6, PCT, and PLT, significantly improved the accuracy of 30-day mortality prediction, with the area under the curve (AUC) increasing from 0.765 to 0.824 (*P*<0.001, Figure 7, Supplementary Table S5). Furthermore, a mediation analysis was performed to examine whether the SOFA score mediated the association between low AT Ⅲ levels and ICU mortality, after adjusting for age, sex, BMI, DM, and malignancy. The total effect of Class 1 on ICU mortality was statistically significant (Estimate=0.111, 95% CI: 0.041 to 0.182, *P*=0.002). The average causal mediation effect (ACME) was 0.025 (95% CI: 0.010 to 0.044, *P*=0.002), indicating that low AT Ⅲ levels were associated with increased ICU mortality partially through higher SOFA scores. The average direct effect (ADE) remained statistically significant after accounting for SOFA and the aforementioned covariates (Estimate=0.085, 95% CI: 0.017 to 0.156, *P*=0.014), suggesting partial mediation. Approximately 22.9% of the total effect of low AT Ⅲ on ICU mortality was mediated by the SOFA score.

## Discussion

This study presented novel dynamic subclasses developed and validated based on AT Ⅲ dynamic trajectory in a prospective multicenter cohort of patients with IAI-induced sepsis. The four AT Ⅲ trajectory subclasses had different clinical characteristics, distinct host responses, and outcomes, which reflect the heterogeneity among patients with IAI-induced sepsis. Persistently decreasing AT Ⅲ levels (Class 1) predicted worse short- and long-term outcomes. This study also confirmed that dynamic trajectories of AT Ⅲ levels were closely associated with changes in SOFA scores.

Changes in AT Ⅲ during sepsis are mainly influenced by liver dysfunction, endothelial injury, and consumption of coagulation factors.^[^^17^^,^^18^^]^ Although AT Ⅲ is primarily synthesized by hepatocytes, a substantial portion of AT Ⅲ binds to the endothelial surface via the heparan sulfate glycosaminoglycan layer. When endothelial injury occurs, the glycocalyx is damaged, thereby affecting plasma AT Ⅲ levels.^[^^19^^]^ Previous studies mostly analyzed the association between AT Ⅲ levels at a single time point and prognosis in sepsis patients. Multiple studies have shown that antithrombin activity is significantly reduced in non-survivors of sepsis.^[^^11^^,^^17^^]^ However, the predictive performance of AT Ⅲ for patient prognosis varied considerably across these studies. An early study in neutropenic patients with sepsis reported a positive predictive value of 0.85 for mortality when AT Ⅲ levels were below 70%.^[^^11^^]^ A prospective observational cohort study reported an AUC of 0.71 for AT Ⅲ predicting in-hospital mortality in sepsis patients.^[^^15^^]^ Single time-point AT Ⅲ levels cannot accurately predict prognosis in sepsis patients. Our results suggest that dynamic changes in AT Ⅲ better reflect the outcomes of IAI-induced sepsis. In the development cohort, both Class 3 and Class 4 subgroups had normal AT Ⅲ activity at diagnosis, but Class 3 showed a rapid decline in the following days, with a 30-day mortality rate of 27.9%, second only to Class 1 (41.6%). Classes 1 and 2 both had initial low AT Ⅲ activity; however, Class 1 experienced a further decline within 3 days, resulting in the highest mortality, whereas Class 2 showed gradual recovery of AT Ⅲ levels and had the lowest ICU and 30-day mortality rates. These markedly different clinical outcomes highlight the necessity of early dynamic monitoring of AT Ⅲ levels in sepsis. Notably, in Class 1, it was observed that the level of AT Ⅲ gradually recovered from <40% on Day 3 to 60% by Day 7, a level that was still below the normal range. The recovery may be attributed to two mechanisms: its resynthesis with the treatment of sepsis and endothelial repair, and diminished degradation by neutrophil elastase after resolution of the acute inflammatory response. Similar AT Ⅲ trajectories and prognostic outcomes were also observed in the validation cohort.

In both the development and validation cohorts, patients with the Class 1 phenotype (low AT Ⅲ, rapid decline) exhibited a highly critical illness. In the development cohort, compared to the other three groups, Class 1 patients showed significant coagulation dysfunction, heightened inflammatory responses, pronounced immunosuppression, and required more organ support. These characteristics likely explain their poor prognosis. Notably, although Class 1 patients had the highest levels of IL-6 and PCT, their WBC counts were paradoxically lower. PCT and IL-6 are common inflammatory markers in sepsis, and their elevation typically reflects a strong inflammatory response to infection.^[^^20^^]^ This contradiction may be attributed to bone marrow suppression caused by excessive inflammation and acquired immune exhaustion. The lower total lymphocyte counts, as well as reduced CD3⁺, CD4⁺, and CD8⁺ T cell counts in Class 1 patients, support this hypothesis. Another prominent feature of this group is severe coagulation disorder, evidenced by significantly elevated ISTH DIC scores, markedly prolonged PT/APTT, and decreased platelet counts. These indicators collectively point to endothelial injury and glycocalyx degradation in sepsis. The coexistence of hypoalbuminemia and hyperlactatemia further supports microcirculatory dysfunction leading to capillary leakage and tissue hypoxia. Furthermore, our study found that dynamic changes in AT Ⅲ trajectories closely correlated with alterations in organ dysfunction. Class 1 patients exhibited the most severe and persistent organ dysfunction, while Class 4 patients maintained higher AT Ⅲ levels and consistently lower SOFA scores.

Notably, although the SOFA score is considered strongly associated with mortality outcomes, the predictive performance of a single SOFA score for sepsis mortality remains controversial.^[^^21^^]^ A prospective, single-center observational study reported good predictive value of SOFA for mortality in older sepsis patients (AUC = 0.802).^[^^22^^]^ Another study found that SOFA outperformed other scoring systems in predicting ICU mortality among sepsis patients (AUC = 0.839).^[^^23^^]^ However, several studies have also suggested that the predictive value of SOFA is limited.^[^^24^^,^^25^^]^ As the AT Ⅲ trajectory partly reflects endothelial function, its incorporation as a Class 1 dynamic trajectory based on SOFA, PCT, IL-6, and PLT significantly improved prognostic prediction for patients with IAI-induced sepsis (AUC = 0.824).

This study has several limitations. First, it included only patients with sepsis caused by abdominal infection, which limits the generalizability of the findings. Due to differences in patient sources and various pathogens, there is a significant discrepancy in mortality rates between the development and validation cohorts. Second, the association analysis did not account for potential confounding effects of interventions such as anticoagulant therapy and blood products on AT Ⅲ levels. Third, the difference in AT Ⅲ testing methods and reagents in different centers may affect the accuracy of AT Ⅲ levels. However, we confirmed that all included centers measured AT Ⅲ activity based on its inhibition of factor IIa (thrombin), with a normal reference range of 70%−125%.

## Conclusions

Patients with IAI-induced sepsis demonstrate heterogeneous temporal AT Ⅲ trajectory phenotypes that correlate with disease severity and mortality. The subclass with an initial low AT followed by a continuous decline exhibits excessive inflammation, coagulation disorders, and severe organ damage, which are closely associated with dynamic changes in SOFA and predict a poor prognosis. Future studies need to focus on stratifying heterogeneous subpopulations with sepsis to conduct precise interventions.

## CRediT authorship contribution statement

**Yuteng Ma:** Writing – review & editing, Writing – original draft, Formal analysis, Conceptualization. **Yini Sun:** Writing – original draft, Supervision, Funding acquisition, Formal analysis. **Chaoyang Wang:** Visualization, Data curation. **Siang Huang:** Visualization, Validation, Data curation. **Qian Gao:** Writing – review & editing, Supervision, Methodology. **Qianyi Xu:** Formal analysis, Data curation. **Mu Sun:** Software, Data curation. **Qiaojie Sun:** Software, Data curation. **Ming Dong:** Writing – review & editing, Writing – original draft, Supervision, Funding acquisition, Conceptualization.

## Appendix Supplementary materials

Image, application 1
