# Supplementary material for: Identifying dynamic antithrombin Ⅲ trajectories to predict clinical outcomes in intra-abdominal sepsis
Source: J Intensive Med. 2026 Jan 20;6(3):284–93. doi: 10.1016/j.jointm.2025.11.006 (PMC13184478; doi:10.1016/j.jointm.2025.11.006)
Supplement: Supplementary file 1 [file mmc1.docx]

**Supplementary Table S1** Fit statistics of LCTM from 1 to 6 subclasses

| **Number of trajectories** | **Loglik** | **AIC** | **BIC** | **Entropy** | **AvePP of each trajectory** | **Proportion of each trajectory** |
| --- | --- | --- | --- | --- | --- | --- |
| 1 | -18451.32 | 36962.66 | 37102.40 | 1.0000 | 1.0000 | 1.0000 |
| 2 | -18801.44 | 37622.88 | 37669.46 | 0.7532 | 0.9241/ 0.9272 | 0.4827 / 0.5173 |
| 3 | -18597.97 | 37225.94 | 37295.81 | 0.7899 | 0.9107/ 0.8984/ 0.9072 | 0.2542 / 0.5481 / 0.1977 |
| 4 | -18531.86 | 37103.73 | 37196.89 | 0.7387 | 0.9085/ 0.8239/ 0.7728/ 0.8975 | 0.2529 / 0.4108 / 0.1425 / 0.1938 |
| 5 | -18483.38 | 37016.76 | 37133.25 | 0.7255 | 0.7911/ 0.9064/ 0.7828/ 0.8016/ 0.8728 | 0.2157 / 0.1887 / 0.1361 / 0.3556 / 0.1040 |
| 6 | -18451.33 | 36962.66 | 37102.40 | 0.7437 | 0.9055/ 0.7932/ 0.8198/ 0.7512/ 0.7972/ 0.8659 | 0.1772 / 0.0436 / 0.3697 / 0.1707 / 0.1438 / 0.0950 |

**Supplementary Table S2** Fixed Effects of four subclasses

| **Variables** | **Coefficient** | **Standard error** |
| --- | --- | --- |
| intercept Class1 | 43.11671 | 1.39287 |
| intercept Class2 | 44.21016 | 1.75993 |
| intercept Class3 | 80.21452 | 3.77455 |
| intercept Class4 | 72.51745 | 1.80100 |
| Day Class 1 | -8.26192 | 0.83400 |
| Day Class 2 | 5.96178 | 1.14087 |
| Day Class 3 | -16.54231 | 2.15973 |
| Day Class 4 | 3.19323 | 1.04226 |
| (Day)^2 Class 1 | 1.62303 | 0.10368 |
| (Day)^2 Class 2 | -0.53650 | 0.14656 |
| (Day)^2 Class 3 | 2.21655 | 0.25356 |
| (Day)^2 Class 4 | -0.54921 | 0.13085 |

**Supplementary Table S3** Clinical characteristics among the four classes of septic patients in the validation cohort

|  | Overall  (*n*=820) | Class 1  (*n*=193) | Class 2  (*n*=310) | Class 3  (*n*=92) | Class 4  (*n*=225) | *p* value |
| --- | --- | --- | --- | --- | --- | --- |
| **Characteristics** |  |  |  |  |  |  |
| Age, years | 67.0 [57.0, 75.0] | 66.0 [56.0, 73.0] | 68.0 [59.0, 75.0] | 67.5 [54.8, 76.5] | 63.0 [55.0, 76.0] | 0.181 |
| Male, n (%) | 493 (60.1) | 117 (60.6) | 186 (60.0) | 58 (63.0) | 132 (58.7) | 0.908 |
| **Comorbidities, n (%)** |  |  |  |  |  |  |
| Hypertension | 325 (39.6) | 65 (33.7) | 114 (36.8) | 50 (54.3) | 96 (42.7) | 0.004 |
| DM | 180 (22.0) | 32 (16.6) | 69 (22.3) | 23 (25.0) | 56 (24.9) | 0.179 |
| Malignancy | 152 (18.5) | 31 (16.1) | 58 (18.7) | 17 (18.5) | 46 (20.4) | 0.722 |
| COPD | 17 (2.07) | 1 (0.52) | 7 (2.26) | 4 (4.35) | 5 (2.22) | 0.155 |
| **Clinical scores** |  |  |  |  |  |  |
| APACHE II score | 19.0 [13.0, 26.0] | 18.0 [13.0, 23.0] | 18.5 [12.2, 25.8] | 18.5 [12.0, 25.0] | 22.0 [13.0, 27.0] | 0.071 |
| SOFA score | 9.00 [6.00, 11.0] | 9.00 [7.00, 12.0] | 9.00 [7.00, 11.0] | 8.00 [6.00, 10.0] | 8.00 [6.00, 11.0] | 0.03 |
| ISTH DIC score | 3.00 [2.00, 4.00] | 3.00 [2.00, 5.00] | 3.00 [2.00, 4.00] | 3.00 [2.00, 4.00] | 3.00 [2.00, 4.00] | 0.052 |
| JAAM DIC score | 3.00 [2.00, 5.00] | 3.00 [2.00, 5.00] | 3.00 [2.00, 5.00] | 3.00 [1.00, 4.00] | 3.00 [2.00, 5.00] | 0.648 |
| **Outcomes** |  |  |  |  |  |  |
| CRRT, n (%) | 162 (19.8) | 40 (20.7) | 59 (19.0) | 18 (19.6) | 45 (20.0) | 0.973 |
| MV duration, hours | 71.5 [32.0, 163] | 88.0 [36.0, 216] | 67.5 [29.0, 157] | 66.0 [39.0, 156] | 64.5 [32.5, 134] | 0.144 |
| ICU LOS, days | 6.00 [3.00, 12.0] | 7.00 [4.00, 13.0] | 6.00 [4.00, 10.0] | 5.00 [3.00, 12.2] | 6.00 [3.00, 11.0] | 0.39 |
| ICU Mortality, n (%) | 92 (11.2) | 37 (19.2) | 25 (8.06) | 11 (12.0) | 19 (8.44) | 0.001 |
| 30-day Mortality, n (%) | 113 (13.8) | 38 (19.7) | 33 (10.6) | 13 (14.1) | 29 (12.9) | 0.039 |

The data are presented as median [interquartile range (IQR)] for continuous variables and count (percentage) for categorical

variables. DM, Diabetes Mellitus; COPD, Chronic Obstructive Pulmonary Disease; CRRT, Continuous Renal Replacement Therapy; MV, Mechanical Ventilation; LOS, Length of Stay.

**Supplementary Table S4** The laboratory variables across subclasses in the validation cohort

|  | Overall  (*n*=820) | Class 1  (*n*=193) | Class 2  (*n*=310) | Class 3  (*n*=92) | Class 4  (*n*=225) | *p* value |
| --- | --- | --- | --- | --- | --- | --- |
| CRP, mg/L | 130 [70.8, 207] | 120 [65.0, 204] | 145 [79.9, 213] | 109 [56.7, 162] | 132 [75.0, 206] | 0.053 |
| PCT, ng/mL | 5.66 [1.56, 18.1] | 5.44 [1.47, 21.6] | 5.88 [2.12, 18.3] | 4.04 [0.96, 13.2] | 5.70 [1.60, 17.1] | 0.301 |
| PLT, ×10⁹/L | 141 [83.0, 198] | 134 [79.0, 200] | 142 [82.8, 190] | 160 [106, 204] | 138 [82.0, 216] | 0.449 |
| LC, ×10⁹/L | 0.61 [0.37, 0.95] | 0.60 [0.36, 0.91] | 0.61 [0.37, 0.93] | 0.65 [0.32, 0.95] | 0.61 [0.41, 1.00] | 0.674 |
| PT, s | 15.4 [13.7, 17.5] | 16.0 [13.9, 18.3] | 15.4 [13.8, 17.3] | 15.0 [13.5, 16.7] | 15.1 [13.5, 17.1] | 0.034 |
| APTT, s | 36.3 [30.8, 43.6] | 38.2 [32.9, 47.6] | 36.4 [31.5, 42.6] | 34.9 [30.0, 40.4] | 34.5 [29.8, 42.5] | <0.001 |
| Fb, g/L | 4.13 [2.91, 5.00] | 3.67 [2.20, 4.88] | 4.01 [2.96, 4.96] | 4.19 [3.19, 5.07] | 4.35 [3.24, 5.28] | <0.001 |
| DD, mg/L | 3.13 [1.60, 6.94] | 3.37 [1.96, 7.32] | 3.19 [1.93, 8.20] | 2.78 [1.12, 5.06] | 2.99 [1.33, 6.68] | 0.067 |
| FDP, mg/L | 15.1 [8.57, 29.1] | 15.2 [8.65, 30.1] | 16.4 [8.41, 28.6] | 13.5 [6.99, 30.9] | 14.3 [9.07, 27.0] | 0.786 |
| WBC, ×10⁹/L | 11.3 [6.87, 17.4] | 11.3 [6.49, 18.2] | 10.6 [5.97, 16.4] | 11.9 [6.99, 17.1] | 12.1 [7.76, 18.4] | 0.278 |
| Lac, mmol/L | 2.20 [1.50, 3.40] | 2.50 [1.67, 3.85] | 2.10 [1.40, 3.10] | 2.40 [1.40, 3.40] | 2.00 [1.40, 3.30] | 0.009 |
| Cr, μmol/L | 102 [69.0, 171] | 97.0 [71.8, 169] | 100 [68.0, 163] | 112 [79.0, 216] | 109 [64.0, 168] | 0.376 |
| TBil, μmol/L | 22.2 [13.7, 44.8] | 23.0 [13.2, 47.2] | 23.3 [14.6, 43.5] | 20.7 [13.4, 32.1] | 20.7 [12.5, 44.8] | 0.372 |

All laboratory variables are presented as median [IQR]. CRP, C-reactive protein, PCT, procalcitonin, PLT, platelet

count, LC, lymphocyte count, PT, prothrombin time, APTT, activated partial thromboplastin time, Fb, fibrinogen, DD, D-dimer, FDP, fibrinogen degradation products, WBC, white blood cell count, Lac, lactate, Cr, creatinine, TBil, total bilirubin.

**Supplementary Table S5 ROC Metrics Predicting 30-day** Mortality in the development cohort

|  | **Sensitivity​** | **Specificity​** | **Youden's index** | **Precision​** | **Accuracy​** | **​F1-score​** |
| --- | --- | --- | --- | --- | --- | --- |
| SOFA | 0.206 | 0.889 | 0.095 | 0.406 | 0.705 | 0.274 |
| Class 1 | 0.460 | 0.860 | 0.320 | 0.547 | 0.752 | 0.500 |
| SOFA + Class 1 | 0.556 | 0.772 | 0.327 | 0.473 | 0.714 | 0.511 |
